# Assessing the impact of gut microbiota and metabolic products on acute lung injury following intestinal ischemia-reperfusion injury: harmful or helpful?

**DOI:** 10.3389/fcimb.2024.1491639

**Published:** 2024-12-02

**Authors:** Qiong Wang, Zi-Hang Yu, Liang Nie, Fei-Xiang Wang, Guo Mu, Bin Lu

**Affiliations:** ^1^ Department of Anesthesiology, Zigong Fourth People’s Hospital, Zigong, Sichuan, China; ^2^ Department of Anesthesiology, Fushun County People’s Hospital, Zigong, Sichuan, China; ^3^ Department of Anesthesiology, The Affiliated Hospital, Southwest Medical University, Luzhou, Sichuan, China

**Keywords:** gut, microbiota, ischemia-reperfusion injury, intestinal ischemia-reperfusion injury, acute lung injury

## Abstract

Ischemia-reperfusion injury (IRI) is a common and clinically significant form of tissue damage encountered in medical practice. This pathological process has been thoroughly investigated across a variety of clinical settings, including, but not limited to, sepsis, organ transplantation, shock, myocardial infarction, cerebral ischemia, and stroke. Intestinal IRI, in particular, is increasingly recognized as a significant clinical entity due to marked changes in the gut microbiota and their metabolic products, often described as the body’s “second genome.” These changes in intestinal IRI lead to profound alterations in the gut microbiota and their metabolic outputs, impacting not only the pathology of intestinal IRI itself but also influencing the function of other organs through various mechanisms. Notable among these are brain, liver, and kidney injuries, with acute lung injury being especially significant. This review seeks to explore in depth the roles and mechanisms of the gut microbiota and their metabolic products in the progression of acute lung injury initiated by intestinal IRI, aiming to provide a theoretical basis and directions for future research into the treatment of related conditions.

## 1 Introduction

Ischemia-reperfusion injury (IRI) is a prevalent and clinically significant form of tissue damage that significantly jeopardizes patient safety (Wu et al., 2018). Ischemia typically results from arterial blockages that reduce blood flow, depriving tissues of essential nutrients and oxygen. Subsequent reperfusion often intensifies this damage and accelerates necrotic processes (Cai et al., 2022). This pathophysiological process has been thoroughly investigated in a range of clinical settings, including but not limited to sepsis, organ transplantation, shock, myocardial infarction, cerebral ischemia, and stroke (Przykaza, 2021; Li et al., 2022; Mao et al., 2022; Pretzsch et al., 2022; Song et al., 2022; Tang et al., 2022). Research across these areas has demonstrated the ubiquitous nature and profound impact of IRI. Particularly noteworthy is intestinal IRI, which occurs frequently in clinical practice and has gained increasing significance due to dramatic changes in the gut microbiota and its metabolic products—often described as the body’s “second genome”—following IRI (Zhu et al., 2010). These alterations underscore the complexity of intestinal IRI and suggest its potential implications for overall health and disease progression. A bibliometric analysis of literature from 2004 to 2022 reveals a substantial volume of research activity in this field, including 1069 articles and reviews. Although the volume of publications on intestinal IRI has stabilized, the growing number of citations reflects the broad recognition and influence of these research findings (Wan et al., 2022).

Intestinal IRI can precipitate secondary damage to multiple organs, including but not limited to the brain, liver, and kidneys. This involves several mechanisms such as activation of microglia, enhanced secretion of exosomes carrying damage signals, and induction of oxidative stress (Zhou et al., 2012; Chen et al., 2020; Zhao et al., 2022). Alterations in the gut microbiota secondary to intestinal IRI can not only exacerbate the primary injury but also adversely affect the function of other organs, including the lungs, liver, and kidneys (Wang et al., 2013; Wen et al., 2020; Chen et al., 2022; Wang et al., 2023). The lungs, particularly vulnerable to damage, face a high risk of acute lung injury (ALI)—a severe, life-threatening condition with significant morbidity and mortality rates (Li et al., 2020). The altered microbiota plays a pivotal role in the pathogenesis and progression of lung injury, not least because the lungs host a distinctive microbial ecosystem which is also influenced by intestinal microbiota changes (Sun et al., 2023; Wang et al., 2023). It is noteworthy that significant shifts in the intestinal microbiome and its metabolic outputs are a hallmark of intestinal IRI, which can further impact the functional outcomes of other organs, deepening the linkage between intestinal IRI and lung injury (Dong et al., 2020; Li et al., 2021; Zhongyin et al., 2022). For instance, mounting evidence has demonstrated that intestinal IRI can precipitate secondary ALI, and modulation of the nuclear factor erythroid 2-related factor 2 signaling cascade and ferroptosis-associated pathways can attenuate intestinal IRI-induced pulmonary dysfunction (Dong et al., 2020). Considering the extensive role of the gut microbiome as an integral part of the human genome in various diseases, advancing research into its specific mechanisms in causing lung injury post-intestinal IRI is crucial for providing significant theoretical insights and opening new avenues for investigation in this domain.

## 2 Intestinal IRI leads to dysbiosis of gut microbiota

During ischemia, the supply of blood and oxygen to the intestines is drastically reduced, significantly impacting the intestinal tissues. In the subsequent reperfusion phase, the substantial accumulation of reactive oxygen species and inflammatory mediators exacerbates the situation, inevitably compromising the integrity of the intestinal barrier. This leads to alterations in the gut’s microecological environment, resulting in changes in both the abundance and diversity of the gut microbiota. These dynamic shifts in microbial communities result in significant alterations in the composition of the microbiota, which may allow microbes and their metabolites to enter the bloodstream, potentially causing dysfunction in multiple organs (Chen et al., 2022).

The changes in the gut microbiota typically manifest in the initial stages following intestinal IRI. Research indicates that within one hour post-IRI, alterations begin in the colonic microbiota, becoming significant by six hours, primarily characterized by increased populations of *Escherichia coli* and *Prevotella*, and proliferation of *Lactobacillus* (Wang et al., 2012). Similarly, the bacterial communities in the ileum undergo early changes during reperfusion and exhibit notable differences after 12 hours, eventually returning to normal (Wang et al., 2013). Deng et al. integrated 16S rRNA and metabolomic analyses to highlight the significant disruption in colonic bacterial composition post-murine intestinal IRI. Notably, there was a significant increase in the relative abundance of *Firmicutes* and *Bacteroidetes*, while the abundance of *Verrucomicrobia* decreased. At the genus level, genera such as *Bacteroides* and *Parabacteroides* distasonis exhibited increasing trends. Metabolomic analysis further revealed that post-IRI, there were disruptions in the expression of genes associated with the biosynthesis and metabolic pathways of secondary metabolites and polysaccharides, alongside changes in metabolites such as capsiate and pravastatin within the microbial community (Deng et al., 2022). These findings underscore that significant changes in the composition of the gut microbiota and their metabolites during the early stages of IRI are critical factors in causing damage to distant organs.

## 3 The dysbiosis induced by intestinal IRI: a double-edged sword with varied effects on different tissues and organs

The early stages of intestinal IRI significantly induce changes in the gut microbiota and their metabolites. These alterations impact not only the intestinal homeostasis but also influence the function of other organs through a complex signaling network, serving as multiple messengers. Specifically, studies demonstrate that the gut microbiota plays a crucial role in suppressing excessive activation of NETosis in neutrophils and enhancing their immune surveillance capabilities, which is essential for maintaining the body’s immune balance and mitigating the inflammatory storm triggered by IRI (Ascher et al., 2020). Subtle pre-IRI changes in the gut microbiota lay the groundwork, enhancing individual susceptibility to IRI through mechanisms including but not limited to the overgrowth of pathogenic intestinal bacteria and abnormal immune system activation. Additionally, a reduction in beneficial microbes, such as those producing short-chain fatty acids, further weakens the gut’s defensive capabilities, increasing susceptibility to IRI (Chen et al., 2022).

Moreover, probiotics like *Lactobacillus* and *Bifidobacterium*, along with tryptophan metabolites, have shown promising potential in improving intestinal IRI. They not only serve as clinical biomarkers for assessing disease severity but also mitigate intestinal IRI through various mechanisms, including enhancing the intestinal barrier function, reducing bacterial translocation, and exerting anti-inflammatory and anti-apoptotic effects, as confirmed by extensive research (Hu et al., 2022; Zhang et al., 2023; Tang et al., 2024). Clinical studies further demonstrate that probiotic use significantly reduces intestinal damage in children caused by medical procedures like extracorporeal circulation (Toritsuka et al., 2024). Additionally, compounds derived from gut microbiota, such as minaprine, pravastatin, and capsiate, have demonstrated their effectiveness in alleviating murine intestinal IRI in experimental models, paving the way for new therapeutic approaches (Deng et al., 2021a; Deng et al., 2021b; Deng et al., 2023).

Notably, gut microbiota metabolites, acting as key intermediaries, significantly influence the extent of damage to other organ functions following intestinal IRI. For instance, succinates, a metabolite from the gut microbiota, have been identified as crucial factors in ALI induced by IRI, with their mechanisms of action progressively being unveiled (Wang et al., 2023). Moreover, intestinal IRI has been demonstrated to trigger other secondary organ dysfunction, particularly affecting the liver and central nervous system (Jing et al., 2012; Chen et al., 2021; Liu et al., 2021). Of particular significance, gut microbiota-derived extracellular vesicles have been shown to modulate the progression of IRI-induced brain injury through multiple mechanistic pathways, encompassing microglial activation regulation, exosome release stimulation, and the TLR4/MyD88 signaling cascade (Zhou et al., 2012; Hsu et al., 2020; Yang et al., 2020; Gao et al., 2024). These findings provide compelling evidence for the pivotal role of the gut microbiota and their metabolic products in the pathogenesis of multi-organ injury following intestinal IRI.

In conclusion, the gut microbiota plays a complex and pivotal role in the onset, progression, and outcome of intestinal IRI, while also being indispensable in the associated damage to other organs. A deeper understanding of the role of gut microbiota and their metabolites in both local and distant organ damage induced by IRI is essential for elucidating damage mechanisms, predicting and diagnosing IRI and its related microbial and metabolic biomarkers, and developing new drugs to treat related injuries.

## 4 High incidence of ALI and its diverse pathogenic mechanisms: the close link between gut microbiota and ALI

ALI, with its high incidence rate, poses significant clinical challenges due to its widespread prevalence and severe consequences. It is reported that each year, approximately 25 per 100,000 individuals are afflicted with ALI, underscoring its frequent occurrence (Parekh et al., 2011). Extensive research indicates that the pathogenesis of ALI is multifaceted, involving multiple levels and pathways. A critical characteristic of ALI is the significant decline in pulmonary gas exchange capability, attributable to both extrapulmonary (indirect) and pulmonary (direct) factors, including lung infections, physical trauma, and other non-cardiac causes. These factors critically impair the integrity of the alveolar-capillary barrier, leading to a cascade of pathophysiological responses. In this context, inflammatory cytokine signaling is aberrantly activated, and immune cells such as platelets and leukocytes, along with a variety of proteins, are released extensively into the alveolar space, intensifying lung inflammation and tissue damage (Zhang et al., 2024). These pathological alterations not only impair respiratory function but may also profoundly affect overall patient health.

Under healthy conditions, a delicate microbial equilibrium known as homeostasis exists between the host and its microbiota, essential for maintaining the host’s physiological functions. Nonetheless, external stressors such as infections, toxins, dietary shifts, and diseases can disturb or disrupt this balance. Gut microbiota dysbiosis can precipitate or accelerate the progression of diseases. Recent research supports a bidirectional communication model between the pulmonary and gut microbiota, with gut microbiota and their metabolic products playing a pivotal role (Wang et al., 2022). Phylogenetically, both the gut and lungs originate from the endoderm and maintain contact with the external environment. In this setting, the epithelial barrier and the microbiota work collectively to defend against pathogens (Enaud et al., 2020). The interplay between the gut microbiota and the lungs, termed the gut-lung axis, is vital for maintaining the immune system’s stability and dynamic equilibrium. Soluble microbial components and their metabolites, transported via the circulatory system, serve as essential mediators of this interaction (Calfee et al., 2015).

Specifically, the gut microbiota can affect ALI progression through various signaling pathways, such as TLR4/NF-kB, AMPK, and T-cell activation regulation (Tang et al., 2021; Wang et al., 2023; Wu et al., 2024). Additionally, the metabolic products of the gut microbiota play a dual role in ALI, exhibiting both beneficial and harmful effects. For example, autoinducer-2 produced by gut microbiota has been shown to exacerbate lung inflammation through the gut-lung axis by promoting the secretion of inflammatory molecules like IL-6, IL-1β, C-C chemokines, and CXCL chemokines in an ALI mouse model when administered intraperitoneally (Zeng et al., 2023). In contrast, another study found that acetate, a metabolic byproduct of intestinal bacteria, could mitigate ALI induced by lung IRI in rats, potentially via the GPR41/43 signaling pathways (Hung et al., 2022). Importantly, the gut microbiota can also facilitate certain therapeutic interventions or medications, impacting ALI progression. For instance, studies have identified that bacterial translocation from the gut may lead to sepsis-associated ALI. The traditional Chinese medicinal formula, Jinhong Decoction, has been found to inhibit this bacterial migration, thus serving in the treatment of sepsis and the resultant ALI (Bao et al., 2023).

Of particular significance, beyond the gut microbiota, the lung-resident microbiome has also emerged as a critical modulator in the pathogenesis of ALI. Specifically, recent investigations have demonstrated that corisin, a microbiota-derived pro-apoptotic peptide originating from the pulmonary microbiome, potentiates inflammatory cell infiltration and accelerates the progression of ALI (D’Alessandro et al., 2023). Concordantly, suppression of corisin biosynthesis has been shown to attenuate acute exacerbation of pulmonary fibrosis (D’Alessandro-Gabazza et al., 2022). These observations underscore the fundamental importance of the pulmonary microbiome and its metabolic products in orchestrating ALI progression. Considering the well-documented reciprocal interactions between the gut and pulmonary microbiota, further elucidation of their intricate interrelationship may provide novel mechanistic insights into the role of the microbiome in intestinal IRI-induced ALI.

These findings underscore the intricate relationship between the gut microbiota and ALI. This interaction may be mediated by the gut microbiota’s influence on specific signaling pathways, regulation of immune cell activation, or direct intervention in ALI progression through metabolic products, and even as mediators for particular therapeutic strategies. These insights affirm the critical role of gut microbiota in the regulation of ALI. Ongoing research is expected to further elucidate the complex interactions between the gut microbiota and their metabolic products with ALI.

## 5 Intestinal dysbiosis following IRI and its impact on subsequent ALI progression

Intestinal IRI constitutes a severe pathological condition with one of its major consequences being the disruption of the intestinal mucosal barrier and abnormal translocation of gut microbiota. In the broad scope of research investigating intestinal IRI-induced ALI, seminal studies have conclusively shown that intestinal IRI serves not only as a pivotal inducer of ALI, but also that lung injury resultant from intestinal IRI can be significantly mitigated by either the elimination or modulation of gut microbiota (Koike et al., 1994; Sorkine et al., 1997). These findings crucially highlight the systemic influence of gut microbiota on lung damage subsequent to intestinal IRI and indicate that diverse microbial subtypes may have distinct roles in this process. Further research is essential to identify microbial subtypes with protective properties.

Additional studies have elucidated the phenomenon of bacterial translocation triggered by intestinal IRI, a process that not only precipitates an overproduction of reactive oxygen species but also instigates the extensive release of inflammatory cytokines and initiates apoptosis. The pervasive dissemination of inflammatory mediators and oxidants through the bloodstream leads to severe systemic inflammatory responses and multi-organ dysfunction, particularly affecting the lungs, liver, and kidneys, with ALI being a frequent complication (Li et al., 2021). Other research has demonstrated that post-IRI, neutrophils and endothelial cells create a unique microenvironment. Within this milieu, the release of oxidants, proteases, and cationic proteins by polymorphonuclear neutrophils during cellular distress compromises pulmonary microvascular integrity and results in severe lung dysfunction. This cascade of reactions not only intensifies lung damage but also promotes the unhindered transport of gut microbiota and their metabolic products to the lungs, establishing a direct pathway for the development of ALI induced by intestinal IRI (Turnage et al., 1994).

**Figure 1:**
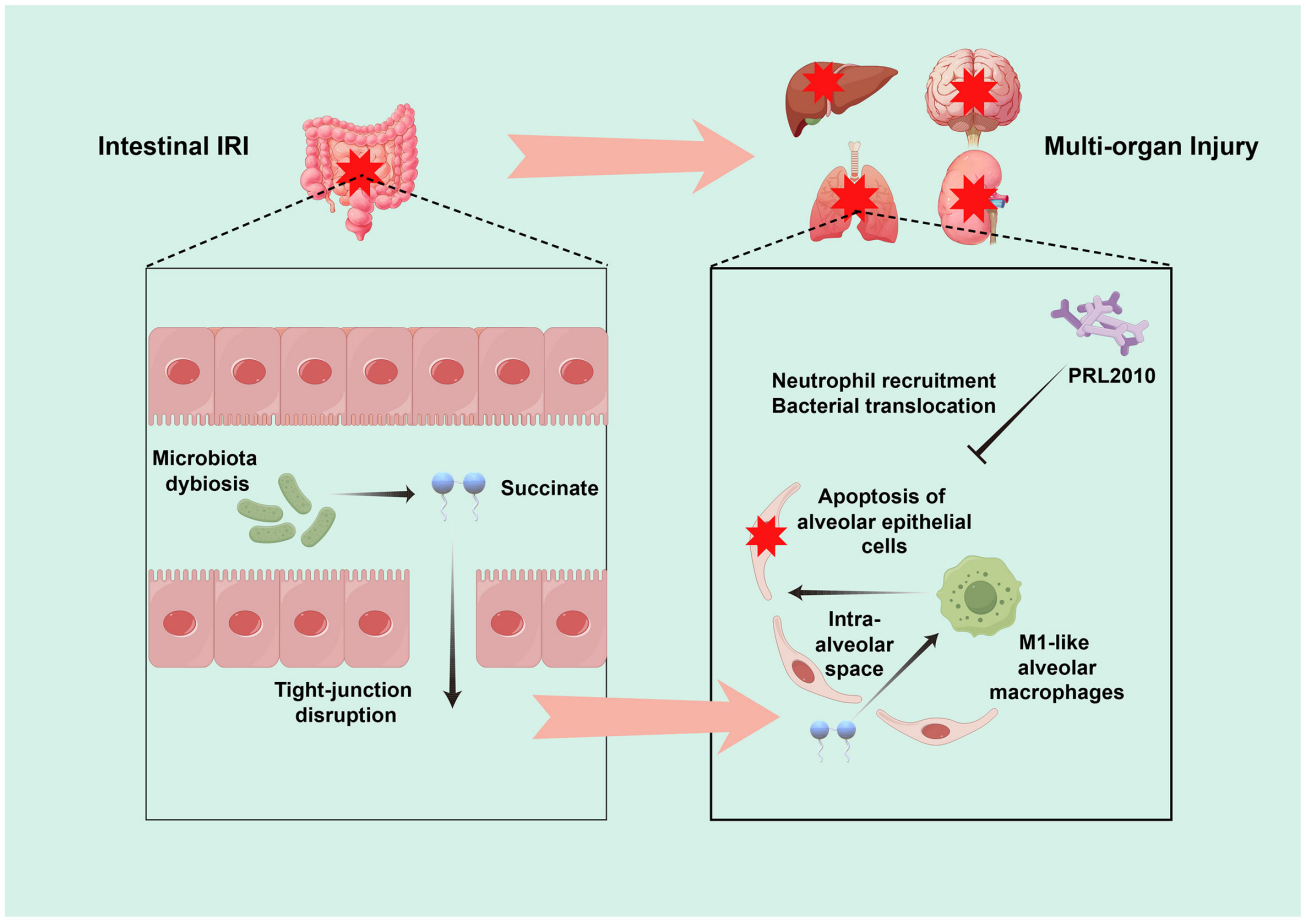
Schematic representation of the pathophysiological mechanisms leading to ALI following intestinal IRI. Post-intestinal IRI, there are pronounced alterations in both the gut microbiota and their metabolic outputs, which contribute to multi-organ dysfunction. A key identified pathway involves a disruption of the equilibrium between the intestinal bacterial populations that produce and consume succinate. This disruption leads to an aberrant accumulation of succinate in the lungs, which fosters the polarization of alveolar macrophages and expedites the apoptosis of alveolar epithelial cells, thereby intensifying pulmonary injury. Moreover, investigation revealed that prophylactic administration of *Bifidobacterium bifidum* PRL2010 significantly attenuated pulmonary neutrophil infiltration and mitigated bacterial translocation in the context of intestinal IRI-induced ALI.

Significantly, Wang et al., through comprehensive research, discovered an abnormal accumulation of a critical gut bacterial metabolic product—succinate—in the lungs following intestinal IRI. This observation underscores a potential metabolic connection between the gut and the lungs. Further analysis revealed that this accumulation correlates with an imbalance in the bacterial populations that produce and consume succinate within the intestine, rather than with the lungs’ metabolic activities, and subsequent experiments affirmed that gut microbiota are the primary contributors to lung succinate levels. During intestinal IRI, succinate not only promotes the polarization of alveolar macrophages but also expedites the apoptosis of alveolar epithelial cells, exacerbating lung damage. *In vitro* and *in vivo* experiments have indicated that downregulating succinate receptors significantly mitigates the adverse effects of succinate, offering prospective targets for clinical interventions. Moreover, Wang et al. also found that clinically, plasma succinate levels significantly correlate with lung injury markers following cardiopulmonary bypass associated with intestinal IRI (Wang et al., 2023). This insight provides not only a novel biomarker for assessing lung injury post-IRI but also forms the basis for designing clinical interventions (**Figure 1**).

Additionally, another study provided new insights into the relationship between intestinal IRI and ALI, demonstrating in a mouse model that pretreatment with the probiotic *Bifidobacterium bifidum* PRL2010 substantially reduced neutrophil recruitment and bacterial translocation in the lungs, suggesting that modulation of gut microbiota could improve ALI prognosis induced by intestinal IRI (Duranti et al., 2018). These studies elucidate the complex interrelations and mechanisms linking intestinal IRI with ALI, offering invaluable references for ongoing research and future clinical applications, thus bridging gaps and directing further exploration of gut-lung interactions.

## 6 Conclusions and perspectives

The recognition of gut microbiota and their metabolic products as a “second genome” has significantly reinforced their pivotal role in the pathogenesis and progression of various diseases. This designation not only reveals the intricate and profound interactions between the gut microbiota and the host but also underscores their potential regulatory roles within disease processes. In particular, intestinal IRI exemplifies a complex pathological process in which disturbed gut microbiota and their metabolic outputs not only impact IRI directly but also exert influence on distant organ functions through intricate mechanisms. This results in complications that include, but are not limited to, lung, liver, and kidney injuries (Wang et al., 2013; Wen et al., 2020; Chen et al., 2022; Wang et al., 2023). Notably, ALI emerges as a particularly significant complication, characterized by high incidence and severity, following bacterial translocation in the context of intestinal IRI (Li et al., 2021). This highlights the critical role of gut microbiota in systemic inflammatory responses and offers fresh insights into the pathological mechanisms underlying intestinal IRI.

Notably, the inappropriate administration of antimicrobial agents in clinical settings presents substantial challenges to gut microbial homeostasis. Specifically, indiscriminate antibiotic usage has been demonstrated to precipitate perturbations in gut microbial community architecture, consequently compromising host physiological functions. Paradoxically, the therapeutic efficacy of certain antimicrobial agents is contingent upon their synergistic interactions with the gut microbiota (Becattini et al., 2016). This complex interplay underscores the imperative for more stringent guidelines governing rational antimicrobial stewardship in clinical practice. Moreover, microbiota-targeted therapeutic interventions have demonstrated considerable promise in ameliorating intestinal IRI-associated pathologies. Specifically, investigations have revealed that probiotic *Bifidobacterium strains* and galactooligosaccharides enhance intestinal barrier integrity in obese adults through competitive interactions with indigenous gut microbiota constituents (Krumbeck et al., 2018). Similarly, fecal microbiota transplantation has exhibited significant modulatory effects on intestinal IRI outcomes (Hu et al., 2022). These observations further validate the fundamental significance of gut microbiota in both basic research and clinical applications. The facilitation of bench-to-bedside translation and implementation of judicious antimicrobial strategies are therefore crucial for optimizing therapeutic outcomes in patients with intestinal IRI.

Additionally, the concept of the intestinal multi-organ injury axis crucially highlights the central role of the intestine in multi-organ damage. Within this framework, the gut microbiota and their metabolic products serve as essential mediators that exacerbate organ function damage. Particularly compelling is the unique microbial ecosystem within lung tissues; the intricate interplay between pulmonary and intestinal microbiota is not only captivating but also of great medical research interest, necessitating further thorough investigation (Whiteside et al., 2021). Moreover, the conventional focus on microbiota dissemination through the bloodstream is complemented by emerging inquiries into whether gut microbiota could also transmit directly via lymphatic pathways. This raises questions about whether such a mode of transmission could alter lymphatic system functions, particularly by impacting immune cell functions, thus playing a mediatory role in lung damage during intestinal IRI (Wang et al., 2022). This avenue of research is not only theoretically innovative but also holds significant clinical application potential, meriting substantial research investment and resource allocation for deeper exploration.

The most direct evidence of the role of gut microbiota in ALI subsequent to intestinal IRI is provided by Wang et al., who have clearly demonstrated that marked changes in gut microbiota and their metabolic products initiated by intestinal IRI can precipitate subsequent ALI. This underscores the central role of the microbiome-mediated gut-lung axis in such scenarios (Wang et al., 2023). To date, the precise roles of the gut microbiota and their metabolites in intestinal IRI-induced ALI remain incompletely elucidated. Intriguingly, accumulating evidence suggests a dichotomous nature of gut microbiota and their metabolic products in intestinal IRI-mediated pulmonary dysfunction, exhibiting both therapeutic and pathogenic properties. This paradoxical phenomenon underscores the intricate mechanistic complexities through which the gut microbiome and its metabolites influence ALI secondary to intestinal IRI.

Although the current evidence base for this research direction is still emerging and requires broader experimental and clinical data for validation, this does not detract from recognizing the vital role that gut microbiota and their metabolic products play in the process of ALI following intestinal IRI. In delving into the complexities of this pathological process, it becomes critically important to explore the dynamic changes in gut microbiota and their metabolic products during intestinal IRI. This endeavor will not only enhance our comprehensive understanding of the pathogenesis of intestinal IRI and its secondary ALI but also provide a robust scientific foundation for developing new therapeutic strategies and optimizing treatment protocols. We believe that ongoing research will enable us to significantly improve clinical outcomes and prognoses for patients impacted by intestinal IRI, ultimately leading to more promising recovery prospects.
